# EEG-based emotion recognition systems; comprehensive study

**DOI:** 10.1016/j.heliyon.2024.e31485

**Published:** 2024-05-18

**Authors:** Hussein Ali Hamzah, Kasim K. Abdalla

**Affiliations:** Electrical Engineering Department, College of Engineering, University of Babylon, Iraq

**Keywords:** Deep learning, Electroencephalography, Brian computer interface, Emotion recognition

## Abstract

Emotion recognition technology through EEG signal analysis is currently a fundamental concept in artificial intelligence. This recognition has major practical implications in emotional health care, human-computer interaction, and so on. This paper provides a comprehensive study of different methods for extracting electroencephalography (EEG) features for emotion recognition from four different perspectives, including time domain features, frequency domain features, time-frequency features, and nonlinear features. We summarize the current pattern recognition methods adopted in most related works, and with the rapid development of deep learning (DL) attracting the attention of researchers in this field, we pay more attention to deep learning-based studies and analyse the characteristics, advantages, disadvantages, and applicable scenarios. Finally, the current challenges and future development directions in this field were summarized. This paper can help novice researchers in this field gain a systematic understanding of the current status of emotion recognition research based on EEG signals and provide ideas for subsequent related research.

## 1 Introduction

**Fig. 1:**
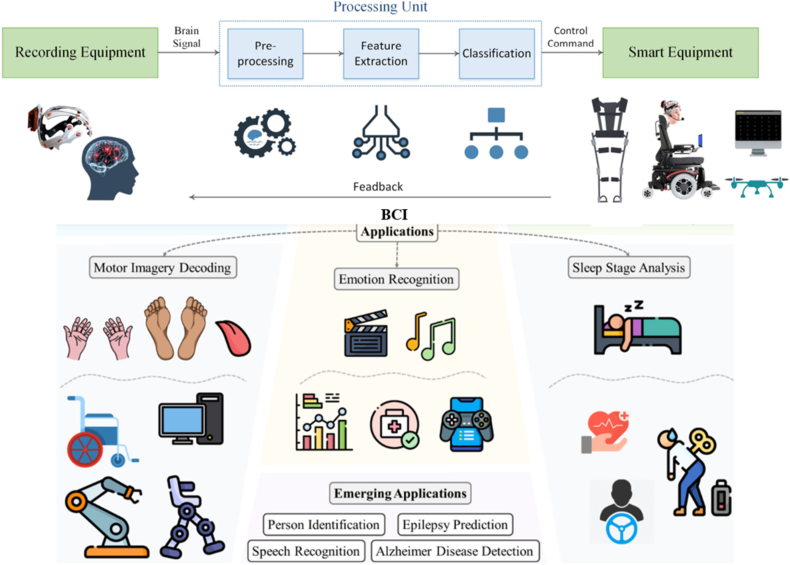
Overview of the BCI and application domain [11,12].

Emotion constitutes a form of amalgamation engendered by individuals when exposed to external stimuli. A favorable emotional state fosters the preservation of both physical and mental health, while prolonged negative emotions exert a significant influence on individuals' mental and physical well-being [1]. To illustrate, persistent melancholy can easily lead to depression, which impairs social function and interpersonal communication and may even jeopardize one's safety. Furthermore, patients suffering from cardiovascular and cerebrovascular diseases are at a higher risk when experiencing intense emotions, such as anger and anxiety. Similarly, drivers who succumb to road rage due to the agitation of other drivers jeopardize not only their own safety but also that of other traffic participants. Therefore, emotions hold a pivotal position in every facet of human life. Consequently, it is of paramount importance to accurately discern emotions [2,3]. Sentiment Analysis is currently a widely employed technique in contemporary times for the analysis of customer reviews, the assessment of popularity in electoral candidates, the identification of hate speech, and comparable applications (see Fig. 1) [4].

At present, the current approaches for recognizing emotions are primarily dichotomized into two aspects. The first approach entails relying on non-physiological information, such as facial expressions, voice tone, and body posture, to identify signals [3]. However, these signals can be deliberately manipulated, making it difficult to obtain representative and authentic signals of emotion. This can impede the accuracy of identifying true emotions and mood states. Alternatively, the second approach employs physiological signals, such as electroencephalography (EEG), electrooculogram (EOG), electrocardiogram (ECG), electromyography (EMG), and galvanic skin response (GSR), for emotion recognition [[5], [6], [7]]. Since physiological signals that accompany emotions are spontaneously generated by the human body's nervous and secretion systems, they are not easily influenced by human factors. By relying on biological emotion recognition through rational signals, physiological signals can more accurately reflect the emotional states of humans. This can lead to more objective and authentic results, which are also more conducive to practical applications [7].

Emotion recognition via EEG is considered one of the most prominent areas of affective computing, which aims to discover and interpret human emotions. Among its applications in the real world [[8], [9], [10]].

1 Increasing the level of interaction between humans and computers by adapting the system's behaviour or reactions according to human feelings. For example, a music player equipped with an EEG device can play the type of music according to the user's current emotions.
2 Improving the level of comfort and mental health by monitoring and regulating people's emotional states. For example, a music therapy site equipped with an EEG device can play music that helps improve the psychology of patients suffering from pain and depression.
3 Developing new models of education and entertainment by creating virtual and interactive environments that respond to human feelings. For example, recognizing and distinguishing human emotions in real time in the form of an avatar in a three-dimensional virtual world.

EEG is a random signal that is discrete in its spatial nature and non-stationary in its character. It has the unique ability to directly record the change in scalp potential. Compared to other physiological signals, EEG can reflect the human condition in a more authentic and dependable manner, especially in a ready state. With the advent of continuous progress in signal processing technology and brain science, EEG signals have gained immense popularity in the field of emotion recognition [13].

The brain network is constructed using data obtained from neuroimaging techniques like electroencephalograms (EEGs) to illustrate the functional relationships between different regions of the brain. The examination of intricate networks can be traced back to the mathematical investigation of networks, which is commonly referred to as graph theory. Contemporary network science has unveiled hierarchical modularity, as well as the presence of rich hubs and clubs, within the organization of the brain network. For electroencephalography (EEG) data, the nodes and links of the graph symbolize electrodes and the relationships among them. Consequently, the neural network extracted from EEG data can be depicted as a connectivity matrix (commonly referred to as an adjacency matrix in graph theory), embodying a graph. Therefore, complex EEG data with multiple channels are transformed into connectivity matrices resembling images, suitable for utilization as input in Convolutional Neural Networks (CNNs) and other computational models [14]. Prior to composing this survey, numerous studies were dedicated to evaluating EEG-based emotion recognition. It is crucial to showcase recent survey papers released within the past three years and elucidate the rationale and urgency behind creating a novel survey paper in this particular research domain. The content of multiple survey documents requires revision. For example, surveys of J. Ma et al. [15] dealing with multimodal sentiment analysis, and R. Vempati et al. [16] they presented a systematic review of automatic emotion recognition from EEG signals using artificial intelligence, X. Wang et al. [17]. they presented a survey of EEG emotion recognition and review benchmark data sets briefly. We believe these surveys should be improved by adding more content about deep learning, different methods to extract EEG features for emotion recognition from four different perspectives, including time domain features, frequency domain features, and time frequency features, and non-linear features. The main contributions of this paper can be classified as follows.

• Identifying emerging trends of AI models tailored for EEG Emotion recognition.
• Review different methods for extracting EEG features (time domain features, frequency domain features, time frequency features, and nonlinear features). We summarized most of the datasets designed for emotion recognition.
• Current challenges and future development directions in this field are summarized.

**Fig. 2:**
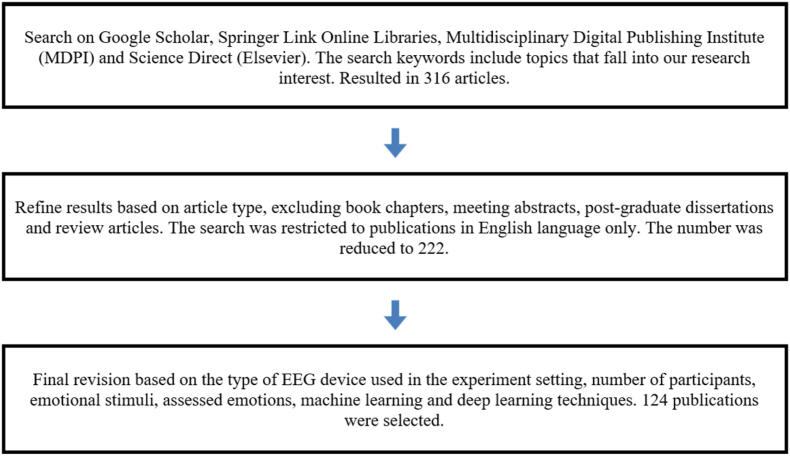
The query for selection and analysis stages of articles.

This research has undertaken a comprehensive examination of BCI emotion recognition systems through a thorough review of the literature. The primary focus of the literature search revolved around widely used citation databases such as (Google Scholar, IEEE Xplore, Springer Link Online Libraries, Multidisciplinary Digital Publishing Institute (MDPI), and Science Direct (Elsevier). Basic search parameters were employed to ascertain subject matters that align with our research focus. The terms and phrases employed in the search encompassed affect or emotion, emotion recognition or classification, and EEG or Electroencephalography. The initial search was further refined to encompass studies published within the past nine years, specifically from 2014 to 2023. This refined search yielded a total of 316 articles, as indicated in (Fig. 2).

## 2 Theories related to EEG emotion recognition research

There are different theories and models that have been proposed to explain how emotions are generated and represented in the brain. In the ensuing sections, we shall succinctly deliberate upon the concept of emotion, models pertaining to the representation of emotion, as well as experiments involving the elicitation or evocation of emotion.

### 2.1 Emotion paradigms

Various brain regions elicit distinct emotions, which can be classified into three categories: reactional, hormonal, and automatic. Emotions, per psychology, are responses to stimuli that involve qualitative physiological alterations and can be categorized into two types: discrete emotion models and multi-dimensional emotion models, as outlined in Ref. [18].

#### 2.1.1 Discrete emotion models

**Fig. 3:**
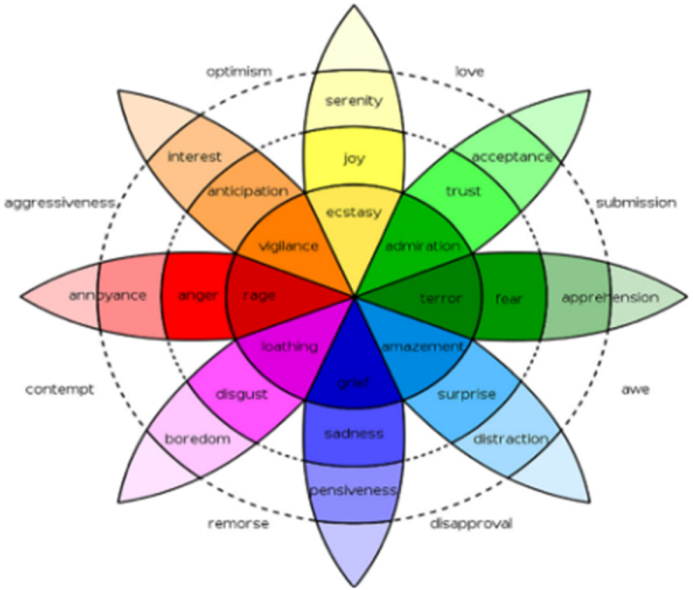
Robert plutchik's wheel of emotions [19].

An emotion quantification model of discrete nature is discussed. Human emotions, as per Darwin's theory of evolution, are considered discrete and have been preserved through natural selection. Approvals of Darwin's viewpoint by Ekman and Plutchik (see Fig. 3) led to the proposal that emotions consist of six or eight fundamental states such as anger, anticipation, fear, sadness, disgust, trust, surprise, joy, etc. which could potentially be expanded to fifteen or more categories. Scholars have further enhanced this model to better quantify emotions. For instance, the Palette Theory suggested that the fundamental emotional states could be likened to Primary Colors, with other emotions being derived by mixing these Primary Colors. For example, the emotions of surprise and sadness can combine to form disappointment. Subsequently, Plutchik introduced an emotion wheel representation method. Furthermore, Parrott and Gerrod proposed a tree-like or hierarchical approach to emotion quantification. Essentially, these quantification models can be integrated into discrete-type emotion quantization models [18,19].

#### 2.1.2 Multi-dimensional emotion models

The multi-dimensional spatial model is grounded on a particular level of specific emotional degrees, which connotes a relationship among diverse emotions [18,20,21]. The identical descriptive emotional conditions might exhibit variances in potencies, for example, fear can be articulated as mild fear or intense fear. Therefore, in order to encapsulate intricate or blended emotions, psychologists have fashioned emotional states in multi-dimensional spatial models, such as two-dimensional and three-dimensional models [18].

##### 2.1.2.1 2D model of emotion space

**Fig. 4:**
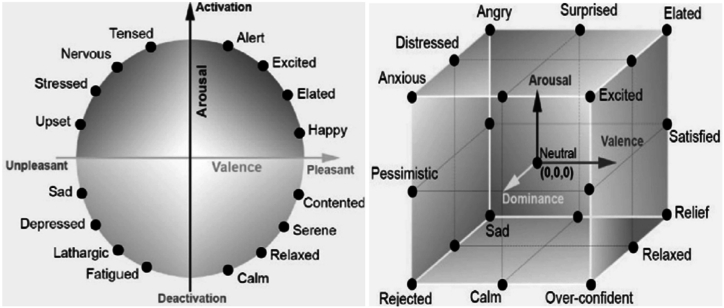
(2D And 3D) Model of emotion space [16,22].

The categorization of a 2-dimensional space of emotion can be accomplished through the assessment of valence and arousal [21]. Valence, which assesses the positive or negative emotions that arise from the perception of emotions, encompasses a spectrum from negativity to positivity and reveals the extent of unpleasant to pleasurable sentiments towards a specific entity. Conversely, arousal measures the intensity of emotions, spanning from a low to high range, and discloses passive or active emotional states that signify the level of intensity of the emotion experienced by an individual. Russell [21] introduced a two-dimensional model, as illustrated in (Fig. 4), wherein happiness is characterized by a positive valence level and high arousal, whereas sadness is linked to a negative valence level and low arousal.

##### 2.1.2.2 3D model of emotion space

Although the 2D model of emotion space is effective in distinguishing between positive and negative emotions, it fails to differentiate between similar emotions that exist within a common quadrant. This limitation necessitates an extension to the model. As an illustrative instance, the emotions of joy and surprise are two affirmative emotional states that possess a substantial degree of valence and arousal, resulting in challenges when attempting to distinguish between them within the confines of the two-dimensional emotion model. To tackle this hurdle, Mehrabian and Russell introduced a three-dimensional emotion model, depicted in (Fig. 4), which encompasses the dimensions of valence, arousal, and dominance (VAD). The third dominant axis spans from a state of passivity to one of dominance, thereby reflecting the extent of control that individuals possess in each emotional state. Within this model, the emotions of joy and surprise can be effortlessly discerned as joy is situated along the high end of the dominant axis, whereas surprise is positioned along the low end of the dominant axis [21].

### 2.2 Acquisition and preprocessing of EEG signals

The electroencephalogram (EEG) is a means of recording electrophysiological indicators of brain activity. It is a summation of extracellular field potentials that arise from the postsynaptic potential in the medium due to the firing activity of numerous neurons in the brain [7]. The EEG signal is broadly classified into two categories: (1) spontaneous EEG, which comprises potential changes that occur spontaneously in the nervous system without any specific external stimulation, and (2) evoked EEG, which involves the imposition of certain sensory stimuli (such as sound, light, image, body sense, etc.), leading to potential changes evoked in corresponding parts of the brain.

**Fig. 5:**
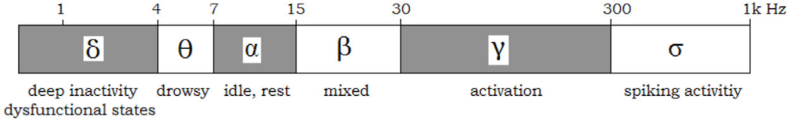
The brain waves: δ, θ, α, β, and γ [23].

The mechanism responsible for the generation of the EEG signal is a complex phenomenon; nonetheless, it embodies a wealth of informative content. This spatially discrete, non-stationary, and time-varying signal exhibits a waveform that is characterized by a scarcity of well-established rules. Therefore, summarizing its behavior is a difficult task. However, from a frequency domain perspective, the EEG signal is perceived as rhythmic, with individual rhythms showcasing specific descriptions that are elucidated in (Fig. 5).

**Fig. 6:**
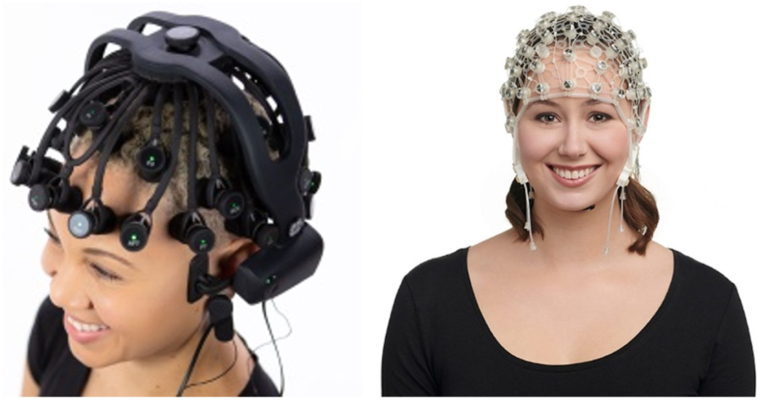
Acquisition of EEG data based on non-invasive equipment.

The acquisition of electroencephalogram (EEG) signals is a fundamental process that involves placing physical electrodes on the scalp. This collection technique can be broadly categorized into two main types: invasive and non-invasive. Invasive methods provide higher accuracy and lower noise, but their use is limited due to safety concerns. Non-invasive methods, depending on the equipment used, can be classified into two main types: dry and wet electrode acquisition equipment. The latter requires conductive media to be placed between the electrodes and the cerebral cortex to reduce resistance interference and increase signal stability, as illustrated in (Fig. 6). However, the use of wet electrode collection equipment is associated with the consumption and wear of the dielectric, leading to a shorter service life, which is not conducive to continuous long-term EEG signal collection. Additionally, the application of thick electrolytes to the scalp can impact the subject's experience. The dry electrode collection apparatus possesses a distinct advantage in that it obviates the need for a conductive medium, thereby reducing the potential discomfort experienced by subjects during lengthy experimentation [6]. This characteristic is highly conducive to facilitating the acquisition of EEG data and the popularization and application of EEG wearable devices. Notwithstanding, the device's sensitivity to the contact between the electrode and the scalp is comparatively low, resulting in a relatively significant degree of interference and weak signal strength. This may subsequently increase the difficulty of feature extraction during subsequent experimentation. Given that both collection devices possess their own strengths and weaknesses, the appropriate selection of either device during the research process should be based on factors such as the length of the experiment and the collection of EEG signals.

**Fig. 7:**
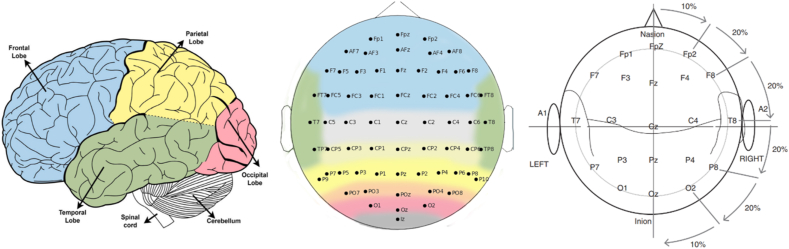
Human brain with standard (10\20) System [24,25].

The EEG collection equipment is equipped with numerous electrodes that serve the purpose of collecting scalp-brain electrical signals. It is noteworthy that the number of electrodes utilized varies among acquisition devices, with 16 electrodes being a common choice. The electrodes used include 16, 32, and 64 electrodes, among others. These electrodes are strategically placed at different locations on the scalp, according to different electrode placement methods including 10–20, 10-10 and 10-5 [17]. These methods help in collecting electroencephalography (EEG) signals from different brain regions. At present, the electrode placement position of the 10–20 system shown in (Fig. 7 and Table 1) is the most widely used. It is important to note that 10 and 20 represent adjacent electrodes, and the actual distance between the skulls is 10 % or 20 %.

**Table 1:** Electrode placement position in (Fig. 7) [24,25].

Brain parts	(Light/Right) of the Hemisphere	Locations of electrodes
F (frontal)	L	Fp1, AF3, AF7, F1, F3, F5, F7, FC1, FC3, FC5
	R	Fp2, AF4, AF8, F2, F4, F6, FC2, FC4, FC6
P (parietal)	L	C1, C3, C5, CP1, CP5, P1, P3, P5
	R	C2, C4, CP2, CP4, CP6, P2, P4, P6
T (temporal)	L	FT7, T3, T5, TP7
	R	FT8, T4, T6, TP8
O (occipital)	L	O1, PO3, PO7
	R	O2, PO4, PO8

## 3 Technical routes of the pattern recognition

**Fig. 8:**
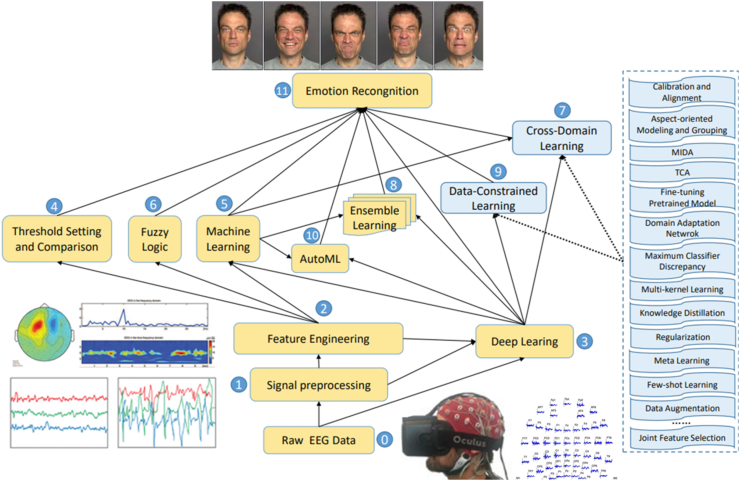
An overview of pattern recognition methods used in modern works, with the various technical approaches clearly highlighted [26].

The study of emotion recognition aligns with the principles of pattern recognition research, whereby emotion categories of target samples are evaluated based on existing data and measurement criteria. A flow chart, depicted in (Fig. 8), presents an overview of the pattern recognition approaches utilized in related works, with diverse technical routes being clearly distinguished. The emergence and advancements of deep learning (DL) in domains such as graph and image processing, as well as natural language processing, have captured the interest of researchers in this field, with existing works demonstrating efficacy [121]. Consequently, this review directs more attention towards DL-based studies, with (Table 2) provide a summary of these technical routes and representative works.

**Table 2:** A summary of recent works on pattern recognition methods using various technical methods in (Fig. 8).

Technical Route	Articles
Threshold setting and comparison 0 → 1→2 → 4→11	[27]
Machin Learning 0 → 1→2 → 5→11	[[28], [29], [30], [31], [32], [33], [34], [35], [36], [37], [38]]
Deep Learning 0 → 1→2 → 3→11	[[39], [40], [41], [42], [43], [44], [45], [46], [47], [48], [49], [50], [51]] [[52], [53], [54], [55], [56]]
Deep Learning 0 → 1→3 → 11 and 0 → 3→11	[47,[57], [58], [59], [60], [61], [62], [63], [64], [65], [66]] [[67], [68], [69], [70]]
Cross domain Learning 3 → 7→11 and 5 → 7→11	[[71], [72], [73], [74], [75], [76], [77], [78], [79], [80], [81], [82], [83], [84], [85], [86], [87]] [[88], [89], [90], [91], [92], [93], [94], [95], [96], [97], [98], [99], [100]] [40,[101], [102], [103], [104], [105], [106], [107], [108], [109]]
Ensemble Learning 3 → 8→11 and 5 → 8→11	[[110], [111], [112]]
Fussy logic 0 → 1→2 → 6→11	[[113], [114], [115]]
Data-Constrained 3 → 9→11	[[116], [117], [118], [119]]
Auto ML 3 → 10→11 and 5 → 10→11	[120,120,121]

## 4 Framework for ER

**Fig. 9:**
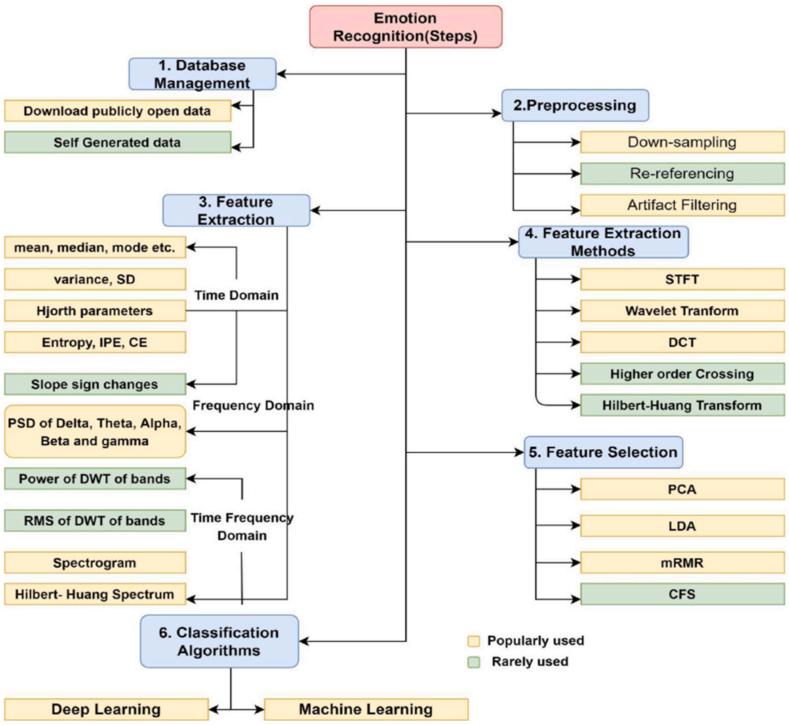
A systematic manual outlining the procedure of recognizing emotions. each stage incorporates the pertinent options [16].

The utilization of EEG signals for emotion recognition can be broken down into several fundamental stages, namely brain electrical signal acquisition, data preprocessing, feature extraction, and classification recognition. Notably, feature extraction can be bifurcated into conventional features and brain network features, constituting two distinct components. This section demonstrates the construction of a system that is capable of emotion recognition through the utilization of an EEG signal. The system operates by extracting relevant features from the signal and employing algorithms to classify these features (Fig. 9). provides a visual representation of the multiple steps involved in each of these procedures.

### 4.1 EEG emotion recognition datasets

**Fig. 10:**
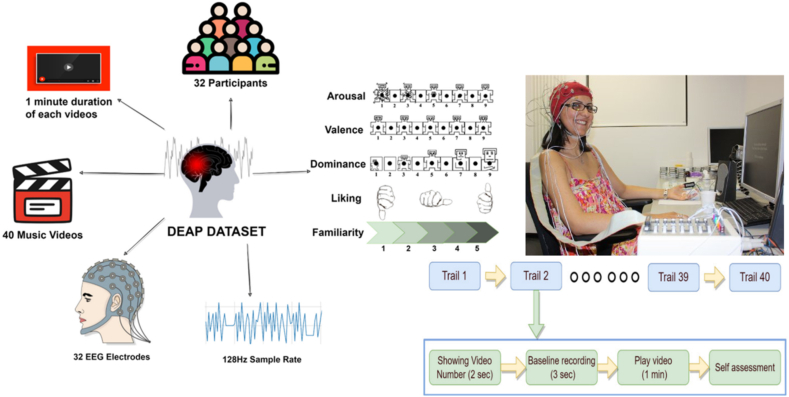
Overview of the benchmark DEAP dataset [123].

There exist two discrete methodologies for acquiring data pertaining to an individual's emotions. The initial strategy encompasses subjecting participants to a wide array of stimuli, encompassing visual cues, auditory signals, audio-visual presentations, and other sensory inputs, encompassing tactile sensations, olfactory perceptions, and gustatory experiences. After this exposure, subjects are prompted to recollect any antecedent emotional states or life occurrences they may have undergone. Recent research indicates that this method is preferred by most scientists, with 26 % of projects utilizing photographs, 23.8 % employing video, 17.5 % incorporating audio, and 22.2 % utilizing a combination of physiological and emotional data. The remaining 10.5 % of projects used emotional data derived from games, performances, or life events. To recognize emotions, EEG data must be collected and recorded. By using the same raw data across multiple studies, it becomes possible to compare results. Consequently, various researchers have aggregated data suitable for research and made it accessible to the public without cost (see Fig. 10) [16,26].

**Fig. 11:**
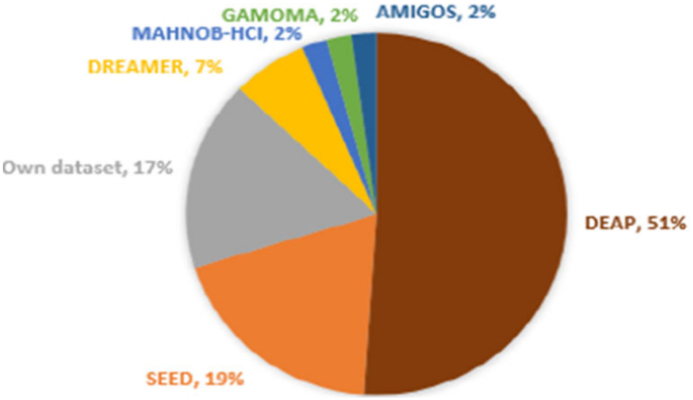
Pie chart of the most widely used open-source dataset [8].

Currently, most of the research concerning emotion recognition is conducted in a tightly controlled experimental environment. A crucial prerequisite for such research is the ability to induce various emotional states in test subjects. Picard et al. [122] categorized the methods used to induce emotions into two distinct types: subject-induced and event-induced. Subject-induced methods rely on the facial expressions exhibited by the subjects themselves or on their ability to recall an event associated with a particular emotion. Event-induced methods, on the other hand, involve the presentation of stimulating material, such as text, pictures, sound, or video clips, to elicit a specific emotional response from the subject. While the Conversation method has been shown to be effective in inducing target emotions, its reliance on the conscious cooperation of the subjects may introduce uncontrollable experimental conditions. To enhance the manageability of the experiment, numerous researchers utilize event-induced techniques to conduct studies pertaining to emotional induction. The employment of video clips as stimuli through the event trigger method amalgamates the benefits of auditory and visual perception of emotional stimulation, thus leading to a more effective induction of emotions. Consequently, the visual frequency segment emotion-eliciting method is extensively employed. Presently, the most utilized mode of emotional induction involves external stimuli such as imagery, music, and videos to evoke diverse emotions amongst subjects. Koelstra et al. [123] conducted a study whereby 32 subjects were exposed to music videos, and their 32-lead EEG signals were recorded during the video, along with their response to the evoked visual frequency valence, arousal, liking, support, and dominance psychological scale, as well as the facial expressions of the first 22 subjects during the emotional videos. Furthermore, they have suggested a multimodal approach for analyzing human emotional state data, thereby creating a data set (DEAP data set) that researchers can employ. The SEED dataset is available for download and utilization by researchers. The Shanghai Jiaotong BCMI laboratory, headed by Professor Lu Baoliang of Tong University, oversaw its provision. The dataset is comprised of carefully selected video clips that aim to stimulate different emotions, namely positive, negative, and neutral. The video materials were presented to 15 Chinese subjects, both male and female, with an average age of 23–27 years old. EEG signals were collected from the subjects while they viewed the 15 Chinese movie clips, consisting of 62 leads [124].

(Table 3) shows The Most Widely Used datasets for emotional recognition. These datasets possess utility for academic investigation, and they have been employed in numerous scholarly inquiries pertaining to the identification and analysis of human emotions (Fig. 11). illustrate the proportion of EEG datasets employed in the identification of emotions, as per the investigations included in this research. The DEAP and SEED datasets are the most utilized, accounting for 51 % and 19 % of the total participation, respectively. A portion of the studies (17 %) utilized their own datasets, which are often not readily accessible. DREAMER, a publicly available dataset, contributed 7 % of the participation in this review. Additionally, our research sample included the datasets (AMIGOS, MAHNOB-HCI and GAMOMA) each of which had a participation rate of 2 %.

**Table 3:** State-of-the-art EEG datasets for emotion recognition.

Dataset\ Ref	Subjects \ (F\M)	Eq\sfreq. Hz	Documented Signals	Stimulu	Targets
DEAP [123]	32 (16\16)	Biosemi Active 2	EEG, EMG, EOG, GSR, Temperature, and Face Video	40 video clips	Arousal, Valence, Dominance
MAHNOB-HCI [125]	27 (16\11)	Biosemi Active 2	EEG, ECG, GSR, ERG, Respiration Amplitude, Skin Temperature, Face Video, Audio Signals, and Eye Gaze	20 video clips	Arousal, Valence, Dominance)
SEED [40,124]	15 (8\7)	NeuroScan \ 1000 H	EEG, Face Video, and Eye tracking	15 video clips	positive, negative, and neutral
DREAMER [126]	25 (11\14)	EMOTIV 128 Hz	EEG, ECG	18 video clips	Arousal, Valence, Dominance
SEED-IV [127]	15 (7\8)	NeuroScan \1000 H	EEG, and EM	24 video clips	(happy, sad, fear, and neutral)
MPED [128]	23 (13\10)	NeuroScan	EEG, ECG, RSP and GSR	28 video clips	(joy, funny, anger, fear, disgust, sad and neutrality)
SEED-VIG [127]	23 (12\11)	NeuroScan \1000Hz	17-channel EEG		vigilance estimation
SEED-V [127]	16 (10\6)	NeuroScan \1000Hz	62-channel EEG	45 video clips	happy, sad, fear, disgust and neutral
SEED-GER [129,130]	8 German (7\1)	NeuroScan	62-channel EEG	18 video clips	positive, negative, and neutral
SEED-FRA [129,130]	8 French	NeuroScan \1000 Hz	62-channel EEG		positive, negative, and neutral
AMIGOS [131,132]	37 (12\25)	Emotiv 128 Hz	14-channel EEG	16 video clips	valence, arousal, control, familiarity, liking
The THU-EP [133]	50 M	NeuSen W32 \ 250 Hz	32-channel wireless EEG	28 video clips	anger, disgust, fear, sadness, amusement, joy, inspiration, and tenderness,
K-EmoCon [134]	32 (12\20) Korean	NeuroSky MindWave	Videos (face, gesture), speech audio, accelerometer, biosignals (2-channel EEG, ECG, BVP, EDA, skin temp.)	16 sessions 10-min	arousal-valence
RCLS [37]	14 (8\6)	NeuroScan \1000 Hz	EEG 64- channel	video clips 4 min	happy, sad, and neutral emotions
The TYUT 2.0 [135]	80 (50\30)	Neuroscan \ 1000 Hz	64-channel, ERP (Event-Related Potential) related to varying durations	Chinese emotional speech stimuli	sadness, anger, happiness, surprise, and neutral ERP (N100, P200,N300)
CMEED [136,137]	37	128 Hz	30-channel EEG device	16 video clips	valence and the arousal dimension
LUMED [96]	11 (4\7)	Neuroelectrics Enobio 500 Hz	visual data (face RGB), (galvanic skin response, heartbeat, temperature), and EEG	video clips (1–2.5) minute	negative valence and positive valence
[138]	29 (7\22)	1000 Hz	128 sensors, covering the forehead and cheeks		
WeDea [139]	30 (12\18)	EMOTIV 128 Hz	14-channel wireless EEG headset	video clips	discrete emotion
DENS [140,141]	(113\548) Indian	250 Hz	128-channel high-density EEG recording technique.	video clips 60s	valence, arousal, dominance, liking, familiarity, and relevance.
ASCERTAIN [142]	58	NeuroSky	EEG, ECG, and GSR sensors	36 video clips (51–128) sec	valence and the arousal dimension
GAMEEMO [143]	28	EMOTIV 128 Hz	14-channel wireless EEG headset	4 computer games 20 min	boring, calm, horror and funny
[144]	20		–	188 food images	positive, neutral, and negative
[145]	44 (23\17)	CLARI TY EEG traveler sensor	32-channel	12 video clips	happy, fear, sad, and neutral
[146]	14	EMOTIV \128 Hz	14-channel wireless EEG headset	IADS and IAPS	arousal, valence, and dominance levels

### 4.2 Preprocessing

During EEG preprocessing, the collected data undergoes transformation and/or reorganization, which occurs between data collection and analysis. This phase enables the grouping of data or the extraction of specific data segments for further analysis without altering the original data. One additional step in preprocessing is the removal of substandard channels, which is accomplished through visual inspection of EEG artifacts. Spatial transformations and temporal filtering are other frequently employed preprocessing techniques [5,13,16,147].

#### 4.2.1 Down-sampling

It is conceivable to reduce the dimensions of electroencephalogram (EEG) data through down-sampling techniques [148]. For instance, within the DEAP database, EEG data sampled at 512 Hz is initially gathered and subsequently down sampled to 128 Hz to facilitate additional analyses. The down-sampled data is an optimal choice for time-dependent computational procedures, storage, or wireless transmission [16].

#### 4.2.2 Re-referencing

Comparing the electrical activity recorded by an electroencephalogram (EEG) electrode to the voltages acquired from other electrodes or the average voltage across multiple electrodes is a widely employed technique. The alteration of the reference point brings about modifications in the shape of the EEG signal, as evidenced by previous investigations. Consequently, accurate registration necessitates the process of re-referencing. Various references, such as the mastoid or CZ, the mean of the two earlobes, or the mean of all electrodes, can be utilized to procure precise recordings [16,149].

#### 4.2.3 Artifact removing

**Fig. 12:**
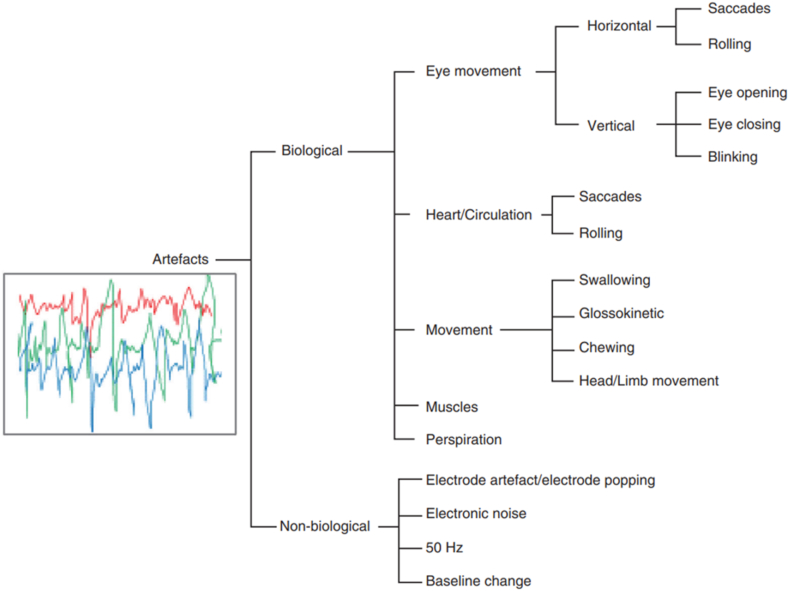
Overview of the artifacts [151].

Internal and external factors both have an impact on the raw EEG data. These factors encompass internal elements like eye blink (EB), eye movement (OM), muscle action, heartbeat, and respiration (refer to Fig. 12). However, it is also necessary to consider external distortions, such as alterations in cable frequency and movement of the head or electrode. Furthermore, the noise originating from the power supply and muscle activity exceeds 40 Hz, whereas the noise stemming from other internal artifacts falls below 4 Hz within the frequency range [16,150].

#### 4.2.4 Band pass filtering (BPF)

It is feasible to eliminate anomalies that have frequencies beyond the designated frequency spectrum of an electroencephalogram signal by administering an appropriate bandpass filter. The ocular reflex occurs at an interval of (2–10) seconds with a frequency range of (0.5–0.1) Hz. The standard pulse rate for an adult range from 60 to 100 beats per minute (1–1.67 Hz). The normal range of respiratory rate, between 12 and 20 breaths per minute, is typically within the frequency range of (0.1–0.34) Hz. However, anomalies caused solely by facial muscle activity or electrooculography influence the electroencephalogram up to a frequency of 20 Hz and are within a frequency range of 0–200 Hz. For this reason, bandpass filtering may eliminate all internal artifacts except for the electrooculography artifact [152]. utilized several bandpass filters, with a frequency range of (4–45) Hz being the most frequently used [152]. The range of (1–100) Hz is moderately utilized, while ranges such as (7–30) Hz, (3–44) Hz, (1–41) Hz, and (3–50) Hz are infrequently utilized [16].

#### 4.2.5 Adaptive filtering

**Fig. 13:**
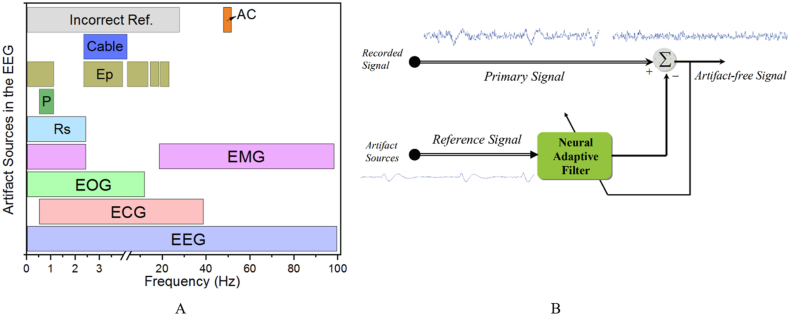
Artifacts removing (A: EEG artifacts and their frequency bands, B: Adaptive filtering) [155].

Several academic inquiries have employed adaptive filtering (AF) to eradicate this form of distortion where the EOG (0.1–200) Hz lies within the range of the authentic EEG signal (4–4) Hz. However, the inclusion of complementary EOG recordings is obligatory to implement adaptive filtering. In the case of AF, both EEG and EOG components are utilized as inputs, and the EOG frequency is subsequently employed to nullify that signal. Certain researchers opt to employ a notch filter at frequencies of 50 Hz and 60 Hz (observe Fig. 13) [16,153,154].

#### 4.2.6 Blind Source Separation BSS

**Fig. 14:**
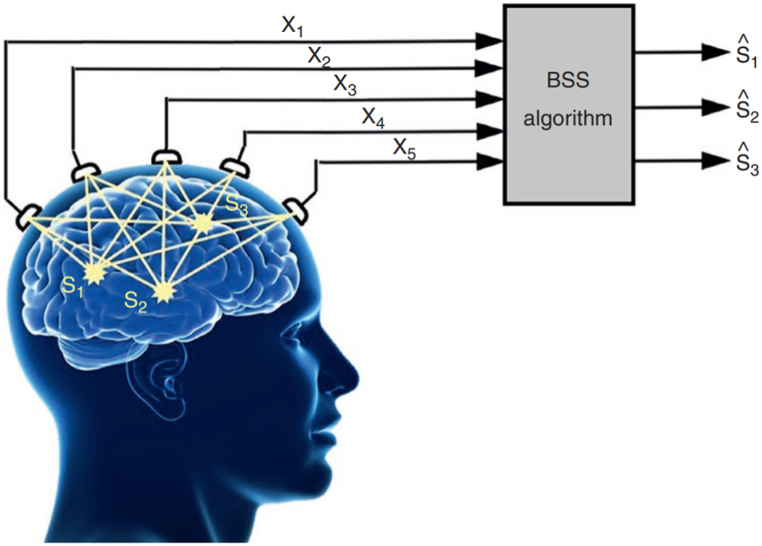
Overview of the BSS concept [157].

Blind Source Separation (BSS) was employed in order to eliminate artifacts and extract precise brain signals from raw EEG signals (see Fig. 14) [156]. The methodologies deliberated upon encompassed ICA, AMUSE, SOBI, and Joint JADE, amongst others. These techniques were implemented to eliminate both internal and external artifacts from the EEG data [156,157].

#### 4.2.7 Independent Component Analysis ICA

The raw EEG signal is a composite one, and through the utilization of the Independent Component Analysis **(**ICA) methodology, distinct and statistically separate features can be discerned. The portions of these independent components that are connected to artifacts are eliminated, and the remaining segments are combined to generate fresh insights. Given that ICA presupposes that the raw EEG data emanates from a group of sources whose sum is not greater than the total number of active channels, it is feasible to pinpoint and segregate numerous distinct signals. To increase the signal-to-noise ratio, the fast ICA [158] technique is often employed in artifact removal [159,160].

### 4.3 EEG feature extraction and selection

In the process of conducting research on emotion recognition using EEG, feature extraction plays a crucial role. Its primary objective is to reduce the dimensionality of EEG data by extracting relevant characteristics that can be used to analyse the emotional state of the subjects [13]. This link in emotion recognition is of utmost importance, as the quality of features directly impacts the performance of the sensory recognition model. Features that have good representation and are highly correlated with emotions are essential for accurate emotion recognition. Currently, there are four main types of EEG signal features that are commonly used: time-domain features, frequency-domain features, time-frequency features, and nonlinear features [13].

#### 4.3.1 Time domain characteristics

The procurement of EEG signals is primarily executed in the temporal domain due to its intuitive and effortless obtainment. The temporal characteristics of EEG signals have been extensively utilized in EEG research, wherein the signal's time domain attributes, namely amplitude, variance, mean, root mean square, zero-crossing analysis, variance analysis, histogram analysis, peak detection, waveform parameter data analysis, and linear prediction, are commonly employed [13]. Kashihara et al. [161] investigated where event-related potentials were obtained via stimulation of subjects. Statistical features, including signal mean and standard deviation, were utilized as EEG features. Tripathi et al. [44] extracted features such as skewness and kurtosis of EEG signals from the DEAP dataset. In their study, deep neural networks (DNN) and convolutional neural networks (CNN) were respectively employed for emotion recognition research in the two dimensions of valence and arousal. The results yielded a positive classification recognition effect. Zhang et al. [162] implemented the amplitude difference between symmetrical electrodes as an EEG features. These features were combined with facial expressions to achieve emotion recognition.

#### 4.3.2 Frequency domain features

The investigation of EEG waveforms over time can be achieved through time-domain analysis. Conversely, the evaluation of EEG waveforms with respect to frequency conditions is accomplished through frequency domain analysis [13]. The fundamental objective of frequency domain analysis is to apply an algorithm that transforms the signal from the time domain to the frequency domain, thereby unveiling the signal's characteristics as they relate to changes in frequency. This approach facilitates a more intuitive observation of the distribution of each rhythm in the EEG. Frequency domain analysis commonly involves dividing the EEG signal into different frequency bands, including delta (0–4 Hz), theta (4–8 Hz), alpha (8–13 Hz), beta (13–25 Hz), and gamma (25–50 Hz) bands, for the purpose of feature extraction. The typical EEG frequency domain features that are extracted include power, power spectral density, and energy quantities. The extraction of these features is primarily based on power spectrum estimation, which is characterized by its ease of calculation, strong adaptability to signals, and clear rationale [13]. Zouridakis et al. [163] acquired electroencephalogram (EEG) signals in five frequency bands, namely delta (0–4 Hz), theta (4–8 Hz), alpha (8–13 Hz), beta (13–25 Hz), and gamma (25–50 Hz). The power spectral density of these bands was then computed as EEG features for emotional recognition. Meanwhile, Gadade et al. utilized fast Fourier transform to transform EEG data from time domain to frequency domain, and band-pass filtering was performed on the theta, alpha, beta, and gamma signals. Power features were calculated for each frequency band and subjected to feature selection using the Relief-F algorithm. Emotional recognition was then conducted in the four dimensions of valence, arousal, domination, and liking, yielding an average accuracy rate of 85 %–92 %. Al-Nafjan et al. [164] employed power spectral density features extracted from EEG in conjunction with deep neural networks to classify emotions. In a similar vein, Li et al. [165] utilized the short-time Fourier transform for time-frequency conversion to calculate the power spectral density features and facial expression features of the theta, alpha, beta, and gamma bands. Subsequently, these features were fused, and the long-short-term memory network was utilized for emotion recognition, resulting in a superior recognition effect.

#### 4.3.3 Time-frequency characteristics

The analysis of the EEG signal is a non-stationary process that necessitates the use of frequency domain analysis. Although the frequency components of the signal can be observed, the temporal appearance of each component remains unknown [5,13]. To address the need a comprehensive understanding of signal frequency changes over time, time-frequency analysis has emerged as an essential tool. This type of analysis accounts for both the temporal and frequency characteristics of the signal, allowing for a more comprehensive depiction of signal changes over time and frequency. As a result, time-frequency analysis can effectively reflect the characteristic information of brain electrical signals. Time-frequency analysis is a widely used technique in signal processing that involves the division of a given time interval into a set of time windows. The resulting signal is then decomposed into a series of smaller processes of equal length, each of which can be considered stationary. The signal is subsequently transformed from the time domain to the frequency domain for each small process, and frequency domain features are extracted. By employing a sliding time window, different time periods can be analysed, allowing for the estimation of the instantaneous frequency and amplitude of the signal at each moment. This provides insight into the duration of emotions. Commonly utilized methods for time-frequency analysis include the short-time Fourier transform (STFT), wavelet transform (WT), and Hilbert-Huang transform (HHT), among others [6,147,166].

The technique known as the short-time Fourier transform was initially proposed by Gabor in 1946. The fundamental concept underlying this approach to time-frequency analysis consists of augmenting the Fourier transform with a window function. Prior to performing the Fourier transform on the signal, a window function is employed under the assumption that the signal remains stable for a brief duration. The window function shifts along the time axis with the aim of segmenting the EEG signal into time units that are equivalent. Subsequently, each of these units is subjected to a Fourier transformation [26,166]:

(1) $$X\left(n,w\right)=\int_{-\infty }^{+\infty }w\left(n-t\right)e^{-i\omega t}dt$$

Where w(n) is the window function, (ω) is the frequency and (t) is the time index. Subsequently, the frequency domain characteristics of each window function are computed, thereby facilitating the derivation of the signal frequency variation over time. In reference to the short-time Fourier transform, it is imperative to meticulously select the appropriate time window length. A narrow time window will result in an insufficient quantity of signals, thereby compromising the accuracy of the frequency domain analysis. Conversely, an excessively wide time window will yield an inadequate time domain resolution [167]. Presently, the optimal time window length for emotion recognition research spans from 1 to 2 s. In 1982, Morlet introduced the wavelet transform as an analytical method of time-frequency transformation. The wavelet transform builds upon and advances the localization concept of the short-time Fourier transform by introducing a variable window that varies with frequency [167]. This approach enhances the immutable time window disadvantage of the short-time Fourier transform. The wavelet transform calculation method is therefore as follows:

(2) $$WT\left(a,\tau \right)=\frac{1}{\sqrt{a}}\int_{-\infty }^{+\infty }f\left(t\right)\psi \;\left(\frac{t-\tau }{a}\right)dt$$

**Fig. 15:**
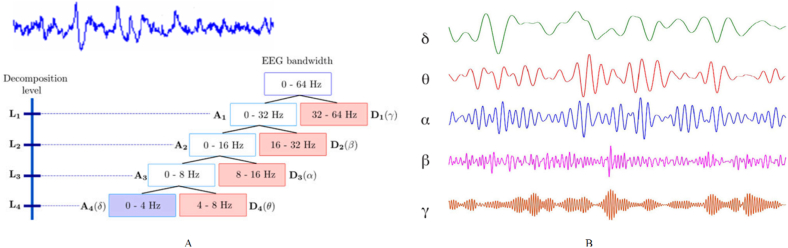
Decomposition process of EEG signal, (A) Illustrations of level decomposition and the corresponding frequency bands of EEG are provided for a sample frequency of 64 Hz. (B) the result of decomposition process: δ, θ, α, β, and γ, five brain waves [24,26].

Where f(t) is the signal, ψ(t) is the mother wavelet, τ is the translation parameter, and a is the scale parameter. Among the parameters, the scaling factor (*a*) holds significant importance. In situations where |*a*| < 1, the basic wavelet undergoes compression, leading to a shift towards higher frequencies. Conversely, when |*a*| > 1, the basic wavelet experiences expansion, causing a shift towards lower frequencies. The translation parameter (*τ*) is utilized to displace the position of the basic wavelet. By applying basic wavelet time window functions of varying widths, signal segments of distinct frequencies can be accurately and efficiently analysed within the time-frequency domain. When the parameters a and *τ* take continuous values, the resulting transformation is referred to as a continuous wavelet transform. However, when these parameters assume discrete values, the transformation is known as the discrete wavelet transform [166,167]. The concept behind the wavelet transform involves modifying the time window function through a variable-length time window truncation transformation based on the short-time Fourier transform. This approach inherits the properties of time-frequency analysis and addresses the limitation of immutable time windows. The degree of variation may change in relation to the frequency of the signal. The wavelet transform comprises two fundamental types: the continuous (CWT), and the discrete wavelet transform (DWT) (see Fig. 15). The Hilbert-Huang transform serves as a nonlinear method for extracting time-frequency features and consists of two primary components: empirical mode decomposition (EMD) and Hilbert spectrum analysis (HSA).

The methods for time-frequency analysis possess both advantages and disadvantages. In accordance with experimental requirements, diverse approaches can be utilized to conduct research on emotion recognition. The selection of an appropriate time-frequency analysis method is crucial for the extraction of time-frequency features. For instance, Murugappan et al. [168] employed the discrete wavelet transform to extract sub-band energy, the proportion of sub-band energy, and the root mean square of alpha-band wavelet coefficients as EEG features. The four emotions in question, namely joy, disgust, fear, and surprise, were categorized based on this method. Chen et al. [169] managed to enhance the accuracy of classification and recognition by utilizing a time-frequency domain emotion feature analysis method, which was obtained from a reconstructed EEG signal source. Further, Chen Meng et al. [166] employed the empirical mode decomposition (EMD) method to analyse the time-frequency of EEG data and to extract the fluctuation index as a feature in order to scrutinize the correlation between the EEG signal and personal emotional state.

##### 4.3.3.1 Time frequency domain features

1.Peak-to-Peak Interval. 2. Mean Square Value. 3. Variance. 4. Mean Value. 5. Skewness. 6. Kurtosis. 7. 1st/2nd Difference. 8. Hjorth Parameter: Mobility, Complexity, Activity. 9. Higher-order Crossing. 10. Maximum Power Spectral Frequency. 11. Power Sum. 12. Maximum Power Spectral Density. 13. Wavelet Energy. 14. Wavelet Entropy. 15. Amplitude and latency of ERPs. 16. Shannon Entropy [13,26].

#### 4.3.4 Nonlinear features

**Fig. 16:**
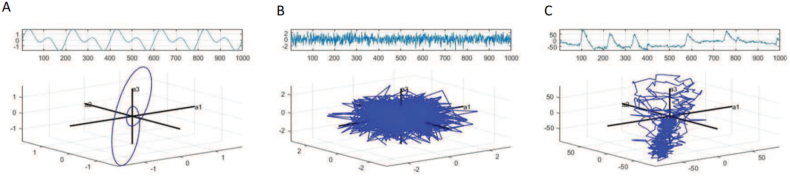
Phase diagram for (A. periodic signal, B. white noise signal, C. EEG signal) [26].

The EEG signal is a time-varying signal that is non-stationary and originates from a complex brain system. Due to its significant nonlinearity and chaos (see Fig. 16), the utilization of linear analysis methods results in the loss of a substantial amount of original EEG signal information. Therefore, nonlinear system and complexity analysis techniques are applied [22]. The emergence of EEG analysis methods corresponds to the needs of the times. The nonlinear analysis method primarily elucidates the relationship between dynamic brain characteristics and various emotional states, investigates alterations in activity within different brain regions during emotional states, and furnishes dependable technical support for related research, such as emotion recognition employing EEG. Among the nonlinear characteristics of EEG signals that are commonly employed are various types of entropy, correlation dimension, fractal dimension, etc. Hosseini et al. [170] conducted an analysis of EEG signals to extract approximate entropy and wavelet entropy features. These features were then utilized for emotion recognition through the application of support vector machine (SVM) algorithms. The resulting accuracy rate was found to be 73.25 %. Similarly, Liu et al. [171] focused on the analysis of six emotions by extracting nonlinear features such as the fractal dimension of EEG signals. Their approach yielded good results, and furthermore, a real-time application system was developed to aid in the treatment of pain, depression, and related symptoms. In a related study, Liu Changyuan et al. [166] extracted frequency band energy, differential entropy, and asymmetric features of EEG signals and utilized support vector machines optimized via the genetic algorithm for emotion classification. The results indicated that the recognition rate for asymmetric entropy features was significantly improved in comparison to traditional features, with an average recognition rate of 88.63 % on the DEAP dataset.

##### 4.3.4.1 Nonlinear dynamical system features

**Fig. 17:**
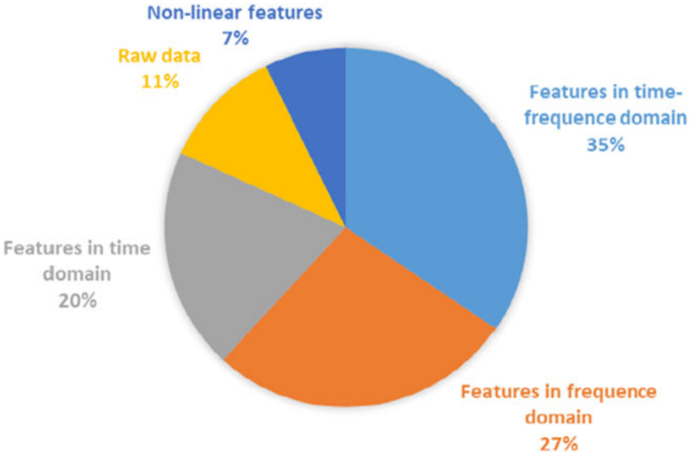
A pie chart depicting the distribution of the domains of the features utilized in the studies.

1. Approximate Entropy. 2. C0 Complexity. 3. Correlation Dimension. 4. Kolmogorov Entropy. 5. Lyapunov Exponent. 6. Permutation Entropy. 7. Singular Entropy. 8. Spectral Entropy. 9. Sample Entropy. 10. Differential Entropy. 11. Fractal Dimension. 12. Hurst Exponent. 13. Lyapunov Complexity. 14. Recurrence Plot: recurrence rate, determinism, entropy, averaged diagonal length, length of the longest diagonal line, laminarity, trapping time, length of longest vertical line, recurrence time of 1st type, recurrence time of 2nd type [22,26].

(Fig. 17) illustrates the proportion of domain usage for the various features incorporated in numerous scholarly articles within this review. The most frequently employed features are those within the time frequency domain, accounting for 35 % of usage. Following closely are frequency domain features, constituting 27 % of usage, while time domain features rank third with a usage percentage of 20 %. Additionally, raw data, in its unprocessed form without any added features, is also employed, accounting for 11 % of usage. Specifically, raw data serves as the input for deep learning algorithms. The utilization of raw data yields satisfactory outcomes, presumably due to the preservation of information and the elimination of the risk of excluding crucial signal features associated with emotion. Moreover, nonlinear features are also employed, amounting to 7 % of usage.

#### 4.3.5 Brain network construction and feature extraction

The brain is a highly intricate and sophisticated system. The manifestation of emotional activity within the brain is not solely attributed to the autonomous functioning of a single cerebral region but rather is the culmination of the interdependent information exchange between numerous brain regions. Traditional approaches to emotion recognition, which are founded upon time-frequency features, tend to disregard the information interplay between cerebral regions. However, in recent years, scholars have increasingly utilized complex network theory in analysing EEG signals with the aim of identifying the cerebral regions that are closely linked to the changes in emotional states of humans. Such endeavours furnish a theoretical foundation for the study and application of EEG signals in the domain of emotion recognition research [13,26]. In an endeavour to scrutinize the prevailing research status of emotion recognition grounded in the brain network, a meticulous exploration was conducted by utilizing the keywords emotion recognition, EEG network, brain network, and emotion recognition. The exploration was conducted via search engines such as Web of Science, Google Scholar, and CNKI. Subsequently, 17 papers based on brain networks were meticulously sieved out. The article, expounding on research pertaining to emotional recognition on the network, meticulously traces the brain network construction methodology and measurement indicators employed in the article.

**Fig. 18:**
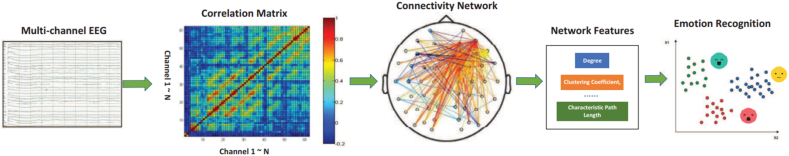
Framework of brain network construction and classification [172].

**Fig. 19:**
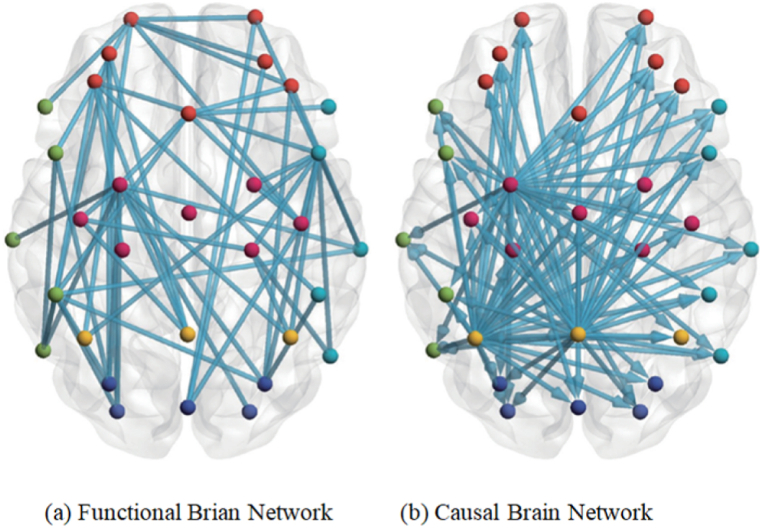
Emotion recognition base on brain network classification [166].

The process of abstracting EEG signals into a physical network within the brain is achieved with the aid of complex network theory. The construction of said brain network entails three distinct steps: the selection of network nodes, the definition of connection edges between nodes, and the selection of appropriate thresholds to facilitate the conversion of the connection matrix to binarization (see Fig. 18). Generally, the collection channel utilized for data gathering is designated as the network node, followed by the selection of an appropriate functional connection method to obtain the functional connectivity matrix. Based on the different construction methods utilized for the functional connectivity matrix, brain networks may be categorized into functional brain networks and causal brain networks [13,166,167]. The functional brain network pertains to the interconnection of various nodes within the brain network and is model-dependent rather than time-dependent. This network is represented as a mutual information model and an undirected network, as illustrated in (Fig. 19 a). The causal brain network, on the other hand, is a distinct functional brain network that shares similarities with the functional brain network concerning data preprocessing and node definition. The primary dissimilarity lies in the causal brain network's ability to demonstrate information flow between different brain regions, thereby transforming the undirected graph of the functional brain network into a directed graph, as depicted in (Fig. 19 b) [166].

##### 4.3.5.1 Brain network features

1. Correlation. 2. Coherence. 3. Clustering Coefficient. 4. Degree. 5. Characteristic Path Length. 6. Local/Global Efficiency. 7. Connectivity Density. 8. Modularity. 9. Closeness Centrality [13,26].

Broadly characterized, cerebral asymmetry pertains to variances in structure or function among homotopic regions within the left and right cerebral hemispheres.

##### 4.3.5.2 Brain asymmetry features

1.Difference Between Channels. 2. Ratio Between Channels. 3. Asymmetry Index (AsI)) [13,26].

### 4.4 ER classifiers

A classifier pertains to an algorithm utilized in the realm of machine learning with the purpose of categorizing data. Presently, numerous scholars employ a diverse range of techniques for classification. Herein, examples of classifiers for the recognition of emotions are provided.

#### 4.4.1 ML based classifiers

**Fig. 20:**
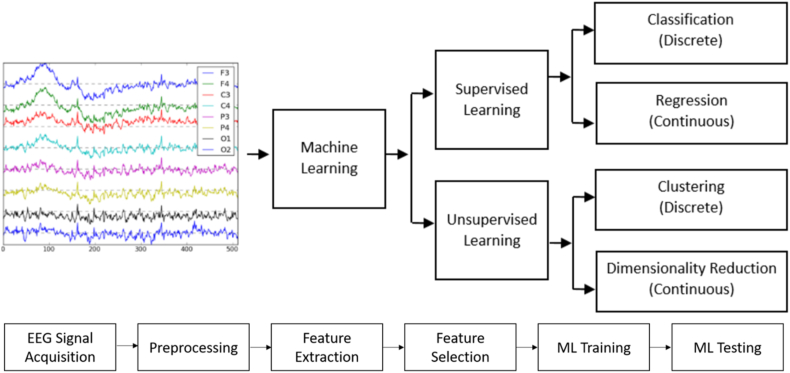
ER based on ML approach with overall steps [187].

In the machine learning studies [29,[173], [174], [175], [176], [177], [178]], SVM are employed for emotion recognition. KNN [179,180], ANN [181,182], RF [31], Decision Tree (DT) [183], MLP [184], RGNN, FG-SVM [185], LSSVM [186], MC-SVM [184] are employed for recognize the human emotions (see Fig. 20 Overview of the ER based on ML mothed).

#### 4.4.2 DL based classifiers

**Fig. 21:**
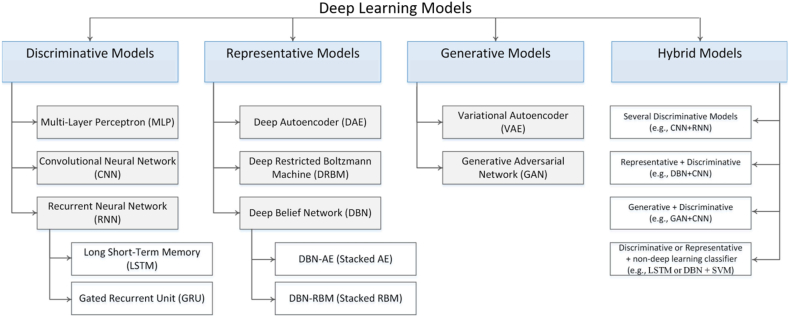
Techniques for classifying of emotion recognition system [16].

Deep learning pertains to a category of machine learning methodologies that leverage multiple layers of information processing stages within hierarchical frameworks for the purpose of pattern classification and feature/relation acquisition. Various subcategories of deep learning algorithms exist, delineated by the specific objectives of the techniques, as illustrated in (Fig. 21). Some advantages of emotion recognition based on deep learning compared to traditional methods [188,189].

1 Deep learning is more capable than traditional methods of dealing with high-dimensional and non-linear EEG data.
2 Deep learning is capable of learning from time-frequency spectra and from raw EEG signals without requiring prior knowledge in this field.
3 Deep learning can take advantage of distributed systems and parallel computing power such as cloud platforms and graphics processing units. To process large-scale EEG signals and train complex models.

**Fig. 22:**
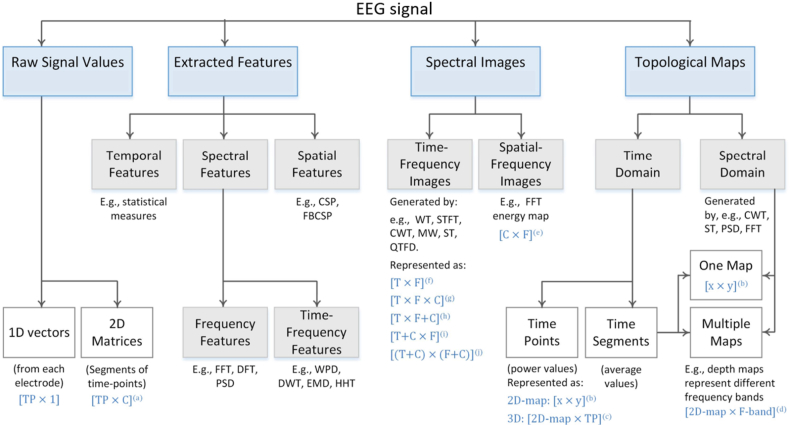
Classification of EEG signal using deep learning methods involves an examination of the taxonomy of various input formulations utilized [16].

The categorization of EEG signal inputs within deep learning frameworks encompasses four distinct categories: derived features, spectral representations, unprocessed signal data, and topographical representations. The selection of input representation was predominantly influenced by the structural design of the deep learning architecture. A visual representation in (Fig. 22) delineates the various input configurations employed in deep learning approaches for the classification of EEG signals. In the deep learning studies [96,190], CNN classifier are used. In Ref. [40], RNN, DBN [40,191], DNN [192], DE-CNN [193], BDAE, CNN employed for CV-CNN [194], H–CNN [45], MC-CNN [195], PC-RNN [196], (RA-CNN) [197] VEn [110], are employed for emotion recognition [198] (Table 4). contains some proposed neural networks inspired by neuroscience, as they contribute to extracting information related to emotions from EEG signals. Some of them can identify implicit connections between channels, which are an important indicator in recognizing emotions. Others can extract discriminatory features and functional connection information simultaneously. A collection of proposed methods to facilitate EEG emotion recognition and provide some explanations for sentiment analysis.

**Table 4:** Proposed neural networks for EEG Emotion Recognition (ER**)** [199].

ER Approach	Structure
ER based on 1D-CNN model	
ER based on 2D-CNN model	
ER based on pretrained model	
ER based on GCNN model	
ER based on CNN-RNN model	
ER based on CNN-AE model	

**Fig. 23:**
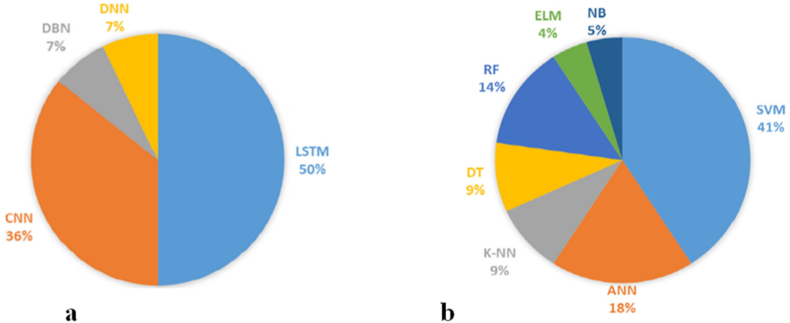
Pie chart of the most widely used (A. Deep learning and B. Machine learning) classifiers.

In (Fig. 23a) depicts a graphical representation showcasing the distribution of the utilization percentage of various deep learning methodologies employed for the purpose of EEG emotion recognition, as discussed within this comprehensive analysis. The utilization of LSTM and its derivatives emerges as the predominant approach, constituting a substantial 50 % of the overall usage. Following closely behind, CNN and its derivatives emerge as the second most favored technique, accounting for 36 % of the examined studies. In contrast, DBN and DNN exhibit significantly lower levels of popularity, each representing a mere 7 % of the total. In (Fig. 23b), Support Vector Machines (SVM) and its derivatives are prevalently utilized, contributing to 41 % of the usage, while the associated kernel functions encompass Gaussian, linear, radial basis functions (RBF), among others. Artificial Neural Networks (ANN) and its MLPNN architecture rank as the second most favored choice, accounting for 18 % of the usage. Random Forest (RF) is selected by 14 % of the researchers. The utilization of K-Nearest Neighbors (K-NN) bears resemblance to that of Decision Tree (DT), with both accounting for 9 % of the usage. A mere 5 % of the researchers opt for Naive Bayes (NB). Extreme Learning Machines (ELM) stands as the least commonly employed technique, with an approximate usage of 4 %. SVM, being a representative of classical machine learning techniques, possesses the advantage of effectively separating classes in a higher-dimensional space through the utilization of diverse kernel functions. Consequently, it has long been favored by many researchers, thus claiming the top spot in this review in terms of algorithm usage frequency. The following (Table 5) shows a list of recent studies focusing on EEG-based emotion recognition.

**Table 5:** Summary of EEG emotion recognition papers using up-to-date methods from 2020 to 2023.

Date\ Ref	Dataset	An up to-date Method	Human Emotion	Results
2020 [200]	Audio-video Clips	ConvNet (LSTM; early and late fusion)	HVHA, LVLA, HVLA and LVHA	accuracy: Early and Late fusion:71.61, 70.17
2020 [201]	DEAP	LSTM	Arousal and Valence	Valence: 94.69 Arousal: 93.13
2020 [202]	Film clips	GPSO-optimized CNN	Fear, happiness, and sadness	Overall Acc: 92.44 ± 3.60
2020 [203]	Own dataset	BiLSTM	Positive, neutral, and negative	Overall Acc:72.83
2020 [63]	SEED	SRU	Positive, neutral, and negative	Overall Acc:80.02
2020 [204]	DEAP and SEED	LSTM	Valence, arousal, and dominance	DEAP: 4 classes: 82.01 Arousal: 85.21 Valance: 84.16 SEED: 90.81
2020 [205]	DEAP	CNN, k-NN, NB, DT	Valence, arousal, and dominance	Overall Acc: 95.20
2020 [58]	DEAP and DREAMR	(RACNN)	Valence, arousal, and dominance	Overall accuracy:96.65 (Valence),97.11 (Arousal)
2020 [206]	Own dataset	LSTM	Happy, fear, anger, sad, Surprise and disgust	Overall Acc: 7.25
2020 [193]	SEED	DECNN	Positive and negative	Overall Acc: 97.56
2020 [207]	AMIGOS	Bidirectional LSTM-RNNs	Valence, arousal, and dominance	Arousal: F1-Score: 61.3, ACC: 73.5; Valence: F1-Score: 58.3, ACC: 67.8
2021 [208]	DEAP, DREAMER, SEED and AMIGOS	CNN + SVM	Arousal and Valence; Positive and negative	DEAP: Arousal:77.7 and Valence: 76.6 DREAMER: Arousal: 90.4 and Valence: 88.2 AMIGOS: Arousal: 90.5 and Valence: 78.4 SEED: 88.5
2021 [209]	DEAP	multi-channel feature fusion	SROCC and PLCC	SROCC: 78.9, PLCC: 84.3
2021 [210]	Own dataset, DEAP\ SEED	LSTM	Disgust, sadness, surprise, and anger Positive, negative, neutral	DEAP: 91.38 SEED: 89.34 Own dataset: 4 class: 94.12 3 class: 92.66
2021 [57]	DEAP	BiDCNN	Arousal and Valence	Subject-dependent Arousal:94.72 Valence: 94.38 Subject-independent Arousal: 63.94 Valence: 68.14
2022 [211]	Own dataset	RNN, LSTM, GRU	positive, negative, and neutral	average Acc: RNN: 95, LSTM: 97, GRU:96
2022 [212]	DEAP	Bi-LSTM	Arousal, valence, and liking	average Acc: valence: 99.45, arousal: 96.87, liking: 99.68.
2022 [213]	DREAMER	LEDPatNet19	arousal, dominance, and valance	valence: 94.58, arousal: 92.86, arousal: 94.44
2022 [214]	DEAP and SEED	ensemble learning	Valence, arousal, and dominance	DEAP average Acc: Arousal: 65.70, valence: 64.22 SEED average Acc: 84.44
2022 [215]	DREAMER and DEAP	(AD-TCNs)	Valence, arousal, and dominance	DEAP average accuracy: Arousal: 64.33, valence: 63.25 DREAMER average Acc: Arousal: 66.56, valence: 63.69
2022 [216]	DEAP and DREAMER	(Bi-CapsNet)	Valence, arousal, and dominance	>25 × reduction on the computational cost, >5 × reduction on the memory usage, <1 % drop on the recognition accuracy
2023 [217]	SEED	(TFSN) (Do-Conv) and (LSTM)	Valence, arousal, and dominance	This model achieves high performance
2023 [218]	SEED and DEAP	EmHM based on (LSTM) and (CNN)	neutral, positive, and negative	Acc 86.50 %, 87.98 % and 91.56 %
2023 [219]	DEAP	EEMD and MEEMD models	Valence, arousal, and dominance	74.3 % for valence and 78 % for arousal
2023 [220]	MAHNOB-HCI	hypercomplex multimodal network	Valence, arousal, and dominance	Arousal: 40.02 ± 1.98 Valence: 43.42 ± 2.57
2023 [221]	Own dataset	(GRU)	Valence, arousal, and dominance	98.85 % (for training) and 91.45 % (for testing
2023 [222]	DEAP	(TF) + (ADDA)	Valence, arousal, and dominance	high performance on DEAP dataset
2023 [223]	SEED and SEED-IV	(PCDG)	Valence, arousal, and dominance	PCDG performed better than other baseline methods but did not have access to the data from target domains
2023 [224]	Own dataset	(CNN) (Resnet18)	Positive, neutral, and negative	93 %. 94.9 %.
2023 [225]	SEED and SEED-IV	(MS-ADRT)	Happy, fear, anger, sad, Surprise and disgust	this methodology offers a significantly enhanced approach to transfer learning for sentiment analysis based on EEG data
2023 [226]	DEAP, SEED, SEED-IV	(RFPN)	Happy, fear, anger, sad, Surprise and disgust arousal and valence	93.56 % (four-class, DEAP) 96.84 % (three-class, SEED) 91.62 % (four-class, SEED-IV)

## 5 Discussion

In the process of channel selection, it is advisable to opt for a lower number of EEG channels due to their ability to diminish the computational time, memory demands, system intricacy, and the time spent on electrode placement. This approach can also mitigate the likelihood of encountering overflow issues that may arise from using unrelated channels. Nevertheless, the selection of a reduced number of electrodes in unsuitable sites could lead to the loss of crucial data. Hence, it is imperative to carefully determine the optimal quantity of electrodes and their suitable placements.

The implementation of channel selection technology to decrease the count of electrodes is presently a topic of significant research interest. At present, there is no universally recognized norm for determining which electrodes are highly correlated with mood states and most accurately reflect changes in mood. The absence of a cohesive framework for the amalgamation of emotion recognition techniques is a crucial issue. In recent years, with the development and application of technologies such as neural networks and deep learning, research methodologies based on EEG for emotion recognition have become increasingly abundant, and many novel approaches have demonstrated successful outcomes. However, to effectively apply EEG-based emotion recognition technology in real-life scenarios, it is essential to establish a unified and comprehensive methodological framework. Disparities in race and users from distinct regions must also be considered. The mechanism of emotional generation necessitates further investigation, including how emotions are generated, the brain regions that are highly correlated with emotional generation, the process of emotional generation, the changes in connectivity between brain regions, the coordination of different brain regions, and the dynamic transformations in the topological structure of brain functional networks during the process of emotional metamorphosis.

The selection of input combinations suitable for leveraging deep learning methodologies hinges largely on the architecture of the deep learning model, as demonstrated in (Fig. 22). The investigations conducted reviewed four distinct input formulations of EEG signals for deep learning networks, as illustrated in (Fig. 22). In CNN models, raw signal data emerged as the most utilized input formulation, with spectral images and extracted features following suit. Nevertheless, CNNs exhibited superior performance when employing topological images as input. Despite the superior performance of topological map inputs in CNN models, the creation of such images is heavily reliant on the quantity of EEG channels. The quality of the topological image is determined by the number of electrodes present, meaning that a greater number of electrodes results in a higher image resolution and consequently, a more accurate representation of spatial information. Hence, all research endeavours proposing the use of topological mapping made use of all EEG channels.

Despite the extensive research conducted on state-of-the-art AI models, there is a need for a more comprehensive comprehension of emotion processes and their neural foundation to develop computational methods for emotion recognition. Additionally, it is crucial to consider psychological, physiological, biological, and brain-inspired cognitive models that are grounded in the principles governing the human brain's function in emotional perception. The prevalent deep learning models merely serve as imprecise mathematical interpretations of brain functions. They have limitations in online learning, small sample learning, and modelling the interaction of information between different brain regions. Biologically inspired approaches are based on the structure of the neocortex and attempt to model the process of how the human brain handles complex information about vision, sound, behaviour, and emotion. In addition, the deep learning model is viewed as a black box that makes it difficult to understand why specific decisions are made. How to solve the problem of poor statistical interpretability should be taken into consideration in future work.

The restricted extent of training data available for tasks involving EEG classification presents challenges to the implementation of deep learning models. These models necessitate a substantial volume of training data in order to converge and generalize effectively to unseen test data. Nevertheless, variations in individuals can result in diverse representations among subjects, complicating the generalization of the model across different subjects. The high-dimensional characteristics of EEG data and its limited accessibility for specific tasks introduce further obstacles to the convergence of these models. A common strategy involves acquiring generalizable features from extensive data through self-supervised learning and subsequently applying them to the relevant task. In this context, we explore the feasibility of training a model on extensive EEG datasets through a self-supervised task and subsequently transferring the acquired knowledge to improve performance in the subsequent task. Notably, large language models (LLMs) have demonstrated remarkable success in natural language processing (NLP) tasks through this methodology. Similar models have demonstrated significant efficacy in various domains such as visual data analysis, medical inquiry responses, and neural pattern examination. Despite the widespread utilization of Large Language Models (LLMs), there exists a noticeable scarcity in endeavours to tailor them for Electroencephalography (EEG) information and implement them in the context of recognizing emotions. The work in Ref. [227] the methodology adopted was influenced by BERT [228] in order to pre-train a modified model on extensive EEG datasets through a self-supervised variational objective. Nevertheless, its applicability to subsequent tasks was found to be constrained. Other paper research employed a pre-trained transformer model known as the generation pre-trained transformer (GPT) model. This model utilizes a transformer architecture with only a decoder component, and it is specifically trained to forecast the subsequent masked token when presented with a series of tokens as input (autoregressive training). In textual tasks, the sentence is disintegrated into tokens, which serve as the input units for the model. The nature of a token may vary, encompassing a few characters or an entire word, depending on the linguistic and contextual factors at play. To apply the GPT model to electroencephalography (EEG) data, the researchers segmented the complete time series into fixed length "segments," treating each segment as an individual token. An EEG encoder was integrated to derive significant features from unprocessed EEG data. Neuro GPT constitutes a fundamental framework encompassing an EEG encoder for extracting spatial and temporal characteristics from EEG data, along with a GPT model that utilizes self-supervision for predicting masked segments. The foundational model underwent pre-training on the TUH EEG dataset [229]. The researchers subsequently fine-tuned the model for a motor imagery classification task with a limited cohort of only 9 subjects. Empirical investigations have indicated that an EEG encoder acquires meaningful features transferable to subsequent tasks. This methodology could be applied for emotion characterization through EEG analysis without the need to eliminate artifacts, to retain valuable emotional cues, present in the EEG data.

## 6 Current challenges

While the suggested deep learning model structures undoubtedly enhance the efficacy of Brain-Computer Interfaces (BCIs), their seamless incorporation is not devoid of obstacles. These obstacles encompass computational effectiveness, variability in data, and interpretability of models. It is imperative to tackle these issues to fully exploit the capabilities of deep learning models in the development and implementation of BCIs.

The necessity of extensive data collections is evident in deep learning models, as their substantial parameters typically demand voluminous datasets to facilitate effective training. Within the realm of Brain-Computer Interfaces (BCIs), the acquisition of sizable, top-notch datasets poses a significant challenge. Despite being prevalent, the DEAP and SEED datasets encounter drawbacks such as inadequate sample sizes, restricted data, and exclusion of certain age demographics.

The computational costs associated with DL models, particularly in their sophisticated setups, impose substantial requirements on computational power and memory distribution. The computational load becomes notably prominent when these models are engaged in the training or adjustment of intricate, high-dimensional electroencephalography (EEG) data. This requirement may impose noteworthy constraints on research teams with restricted resources, inhibiting them from fully leveraging the potential of these models.

The interpretability of the model is a crucial element in the field of deep learning, which has been extensively investigated. Despite their capacity to handle vast amounts of data and produce outstanding outcomes across various tasks, these models can exhibit high complexity, posing challenges in comprehending the basis of their predictions. This lack of clarity in the decision-making process can have significant implications, particularly in domains like healthcare, where EEG data are pivotal, and misinterpretations can result in erroneous medical interventions. Hence, there is a pressing need to enhance the transparency and interpretability of such models, ensuring that the rationale behind their decisions is accessible and comprehensible.

Transfer learning and domain adaptation are essential topics to consider. The enduring success of deep learning models across various subjects and devices poses a significant obstacle within the realm of Brain-Computer Interface (BCI). The act of training a model using data from a specific cohort of subjects or a specific device could lead to a decline in effectiveness when implemented on an alternate cohort or device. The existence of this disparity can be ascribed to numerous factors, with the most noteworthy ones being: (variability between subjects, variability between experiments, inconsistent electrode placement, device-specific biases, and environmental noise).

## 7 Future directions

**Fig. 24:**
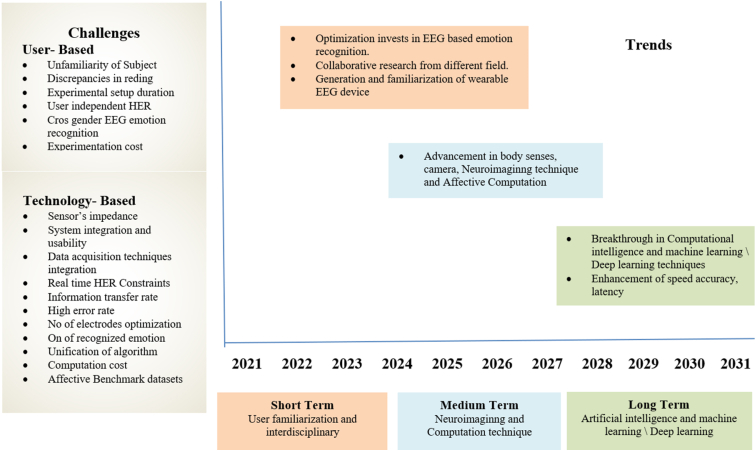
Recognize the challenges and potential advances in research on EEG-based emotion recognition [230].

The utilization of deep learning architectures in the realm of communication interfaces, specifically brain-computer interfaces (BCIs), is a continuously developing area with considerable promise. Through technological advancements and improvements in research approaches, various pathways are being explored that offer the potential for improved incorporation of deep learning models (transformer, Graph Neural Network and LLMs) into BCIs (Fig. 24). illustrates the prospective directions for research, challenges, and advancements in EEG-based recognition of human emotions as perceived by us. The elucidation encompasses numerous challenges related to emotion recognition within the context of Brain-Computer Interface (BCI), which is commonly categorized into two domains: technology-oriented and user-centric. Furthermore, it delineates the potential impact of technological progress and trends on the realm of research concerning EEG-based recognition of human emotions. The identification of emotions within the domain of BCI continues to pose a significant challenge, necessitating further investigation and empirical studies. The imperative task of senior researchers in formulating a dependable system for classifying emotions remains essential to facilitate seamless interaction between individuals and automated systems.

## 8 Conclusion

In this investigation, a comprehensive overview is provided on deep learning networks utilized for classifying EEG signals. Unlike conventional approaches, deep learning has the capability to autonomously extract intricate high-level latent features by leveraging the deep architecture of unprocessed EEG data. Examination is conducted on the preprocessing techniques, input representation, deep learning methodologies, and network structure, followed by an assessment of the efficiency of cutting-edge deep learning techniques.

Training a model using data from a specific cohort of individuals or a specific apparatus could lead to diminished efficacy when implemented on a separate cohort or apparatus. This variation can be ascribed to numerous contributing elements.

• Variation between subjects: Each person's brain exhibits distinct characteristics and patterns, attributable to variations in anatomy, functional organization, and neural plasticity. These differences can manifest in unique electroencephalographic (EEG) signal patterns, particularly evident during comparable cognitive tasks.
• Variation between trials: An individual's brain signals can exhibit variations between various trials and sessions, attributable to factors like fatigue, attention levels, and the time of day.
• Inconsistency in electrode placement: Minor variations in the positioning of electrodes during different sessions or among different individuals may result in variability. This variability may be attributed to factors such as human error, variations in cranial morphology, or differences in hair thickness.
• Device-specific biases: Various EEG devices might possess distinct calibration configurations, sampling frequencies, or signal-to-noise ratios, all of which could result in inconsistencies in the collected data.
• Environmental noise: External variables, such as ambient light, noise, or room temperature, have the potential to influence the neural activity of an individual, which can subsequently introduce complexities when attempting to compare across different individuals.

Meeting the Challenge: Meeting this challenge necessitates the utilization of advanced methodologies capable of standardizing and accommodating the intrinsic variables at play, thereby ensuring the resilience and applicability of BCI models beyond specific data sets. Techniques for domain adaptation, which involve modifying a model initially trained within one domain to effectively operate within a distinct yet interconnected domain, offer a promising avenue for exploration. Additionally, enhancing performance through fine-tuning with more focused datasets or implementing methodologies like meta-learning, which enable models to swiftly acclimate to new tasks, can prove advantageous. in summary, emotions have a significant impact on various aspects of human social life, behavioural regulation, and mental health. The research conducted on emotion recognition has paramount theoretical implications and application value. As EEG acquisition technology and signal processing technology continue to advance, there has been a surge of related research on emotion recognition via EEG signals, which has yielded widely recognized research outcomes. Building on the existing research findings, we aim to optimize the emotion recognition methodology based on EEG signals, identify brain regions and frequency bands that are closely associated with emotional states, and develop a dependable, non-user-dependent wearable emotion recognition device. The application value and societal benefits of electro-emotion recognition research represent crucial endeavours for the future.

## Funding

This research received no external funding.

## CRediT authorship contribution statement

**Hussein Ali Hamzah:** Investigation, Writing – original draft, Writing – review & editing. **Kasim K. Abdalla:** Supervision.

## Declaration of competing interest

The authors whose names are listed immediately below certify that they have NO affiliations with or involvement in any organization or entity with any financial interest (such as honoraria; educational grants; participation in speakers’ bureaus; membership, employment, consultancies, stock ownership, or other equity interest; and expert testimony or patent-licensing arrangements), or non-financial interest (such as personal or professional relationships, affiliations, knowledge or beliefs) in the subject matter or materials discussed in this manuscript.

## Abbreviations

ER: emotion recognition
CNN: Convolutional Neural Network
RNN: Recurrent Neural Network
DBN: Deep Belief Network
BDAE: Bimodal Deep Auto Encoder
CV-CNN: Computer Vision
H–CNN: Hierarchical Convolutional Neural Network
Ven: Voting Ensembles
SVM: support Vector Machine
KNN: K Nearest Neighbor
ANN: artificial neural network
RF: Random Forest
DT: Decision Tree
MLP: Multi-Layer Perceptron
RGNN: Regularized Graph Neural Network
FG-SVM: Fine Gaussian Support Vector Machine
LSSVM: Least Square Support Vector Machine
MC-SVM: Multi-Class Support Vector Machine
LSTM: long short-term memory
GPSO: gradient-priority particle swarm optimization
BiLSTM: Bidirectional LSTM
SRU: RACNN: Regional- Asymmetric Convolutional Neural Network
DECNN: BiDCNN: LEDPatNet19: AD-TCNs: adversarial discriminative temporal convolutional networks
Bi-CapsNet: binary capsule network
TFSN: Time-Frequency-Spatial Network
Do-Conv: deep over-parameterized convolutional layer
EmHM: Emotion Hybrid Model
CNN: convolutional neural network
EEMD: MEEMD: ensemble median empirical mode decomposition
GRU: Gate Recurrent Unit
KNN: K-Nearest Neighbor
ANN: Artificial Neural Network
ICBRC: unconventional improved Cluster-based region Classifier
TF: transformers
ADDA: adversarial discriminative domain adaptation
PCDG: prototypical contrastive domain generalization framework
ACRNN: attention-based convolutional recurrent neural network
MS-ADRT: multi-source attention-based dynamic residual transfer
RFPN: residual feature pyramid network
